# A multi-chamber microfluidic intestinal barrier model using Caco-2 cells for drug transport studies

**DOI:** 10.1371/journal.pone.0197101

**Published:** 2018-05-10

**Authors:** Hsih-Yin Tan, Sofie Trier, Ulrik L. Rahbek, Martin Dufva, Jörg P. Kutter, Thomas L. Andresen

**Affiliations:** 1 Technical University of Denmark, Department of Micro and Nanotechnology, Ørsteds Plads, Lyngby, Denmark; 2 Technical University of Denmark, Center for Nanomedicine and Theranostics, Ørsteds Plads, Lyngby, Denmark; 3 Biomedical Institute for Global Health Research & Technology (BIGHEART), National University of Singapore, Singapore; 4 Global Research, Novo Nordisk A/S, Maaloev, Denmark; 5 Department of Pharmacy, University of Copenhagen, Universitetsparken 2, Copenhagen, Denmark; Hungarian Academy of Sciences, HUNGARY

## Abstract

This paper presents the design and fabrication of a multi-layer and multi-chamber microchip system using thiol-ene ‘click chemistry’ aimed for drug transport studies across tissue barrier models. The fabrication process enables rapid prototyping of multi-layer microfluidic chips using different thiol-ene polymer mixtures, where porous Teflon membranes for cell monolayer growth were incorporated by masked sandwiching thiol-ene-based fluid layers. Electrodes for trans-epithelial electrical resistance (TEER) measurements were incorporated using low-melting soldering wires in combination with platinum wires, enabling parallel real-time monitoring of barrier integrity for the eight chambers. Additionally, the translucent porous Teflon membrane enabled optical monitoring of cell monolayers. The device was developed and tested with the Caco-2 intestinal model, and compared to the conventional Transwell system. Cell monolayer differentiation was assessed via *in situ* immunocytochemistry of tight junction and mucus proteins, P-glycoprotein 1 (P-gp) mediated efflux of Rhodamine 123, and brush border aminopeptidase activity. Monolayer tightness and relevance for drug delivery research was evaluated through permeability studies of mannitol, dextran and insulin, alone or in combination with the absorption enhancer tetradecylmaltoside (TDM). The thiol-ene-based microchip material and electrodes were highly compatible with cell growth. In fact, Caco-2 cells cultured in the device displayed differentiation, mucus production, directional transport and aminopeptidase activity within 9–10 days of cell culture, indicating robust barrier formation at a faster rate than in conventional Transwell models. The cell monolayer displayed high TEER and tightness towards hydrophilic compounds, whereas co-administration of an absorption enhancer elicited TEER-decrease and increased permeability similar to the Transwell cultures. The presented cell barrier microdevice constitutes a relevant tissue barrier model, enabling transport studies of drugs and chemicals under real-time optical and functional monitoring in eight parallel chambers, thereby increasing the throughput compared to previously reported microdevices.

## Introduction

Covering the inner wall of the small intestine is a single layer of epithelial cells that forms a rate-limiting barrier for the absorption of drugs. Numerous experimental models have been developed to predict intestinal permeability—including *in situ* isolated perfused intestinal systems [1–4]. However, the use of animal models is time consuming, labour intensive and costly. Furthermore, animal models also raise ethical issues and are often not able to accurately predict the results in humans [5]. Culturing and differentiation of epithelial cells derived from the intestine can provide relevant *in vitro* models for prediction of drug absorption in humans [6,7]. Caco-2 cells constitute a gold standard of intestinal model when cultured under specific conditions, i.e., grown on Transwell permeable filter supports, the cells will form a monolayer [8]. They will further spontaneously differentiate and proliferate, thus exhibiting many features of the small intestinal villus epithelium [9,10]. Some of the most prominent features of Caco-2 cells cultured in this way are the formation of brush border microvilli [10] on the upper side of the cells, development of intercellular tight junctions [11], and the presence of various metabolic enzymes present in the intestinal epithelium [10,12]. Due to the formation of a tight monolayer of Caco-2 cells, this provides a physical and biochemical barrier to the passage of ions and small molecules through the Caco-2 cell layer. Therefore, it is one of the most well-established human intestinal epithelial cell lines and has been extensively used as an *in vitro* intestinal model for pharmaceutical studies, e.g., ADME-Tox (adsorption, distribution, metabolism, excretion, and toxicology) studies. About three weeks are required for Caco-2 cells to fully differentiate and form confluent and tight monolayers in Transwell inserts [8]. However, this *in vitro* model does not incorporate the continuous fluid flow nor the fluid shear stresses experience by epithelial cells *in vivo* (reported as 1 to 5 dyn/cm^2^ [13] depending on location).

By using micro total analysis system technology, various functional microfluidic systems can be developed to provide integrated microenvironments for cell maintenance, continuous perfusion and real-time monitoring of cells. Several groups have reported on the design and fabrication of polydimethylsiloxane (PDMS) based microdevices for Caco-2 cell culture [14–18]. Kim et al. have reported that with the combination of peristaltic motion and fluid flow in the microfluidic device, Caco-2 cells displayed intestinal villi-like structures with physiological growth up to several hundreds of microns in height, as well as increased expression of intestine-specific functions, including mucus production [17]. However, the reported microfluidic devices are only capable of culturing one set of Caco-2 monolayers for analysis at any one time. In biological cell analysis studies or drug transport studies across Caco-2 monolayers, or other model tissue barriers, it is highly desirable to investigate different conditions in the same experimental system in a high throughput manner [19–22]. Scaling up the number of cell culture microchambers on the microfluidic chip provides the possibility for analyzing more than one sample in parallel under controlled conditions, therefore allowing for controlled parameter comparisons. Recently, Trietsch et. al. reported on utilizing the commercial OrganoPlate platform for culturing 40 membrane-free ‘gut-tubes’ in a 384-well microtiter plate format [23]. In this planar platform, there are two compartments namely the lumen of the ‘gut-tube’ and the ‘blood-vessel’. These two compartments were separated by an extracellular matrix (ECM) gel. Although PDMS is an excellent material choice for fabricating microfluidic devices for cell culture, it may pose some challenges in studies that involve chemicals and drugs. Studies have shown that PDMS has a tendency to absorb small hydrophobic molecules [24–27] and this may compromise accurate measurements of drug efficacy and toxicity [26–28]. Alternative materials such as polymethylmetacrylate (PMMA) [29] and physiologically relevant materials have been explored and reported for organ-cell culture in microfluidic devices [30].

**Fig 1:**
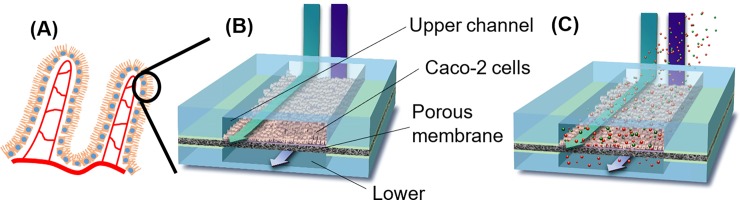
Schematic illustrations of the thiol-ene based microfluidic chip for intestinal transport studies. (A) Cross-sectional view of human intestinal microvilli. (B) Microarchitecture of one microchamber on the thiol-ene microfluidic chip consisting of upper and lower channel cell culture chambers separated by an ECM coated Teflon membrane. (C) Co-administration of an absorption enhancer to increase Caco-2 monolayer permeability.

To develop an alternative *in vitro* model of the human intestines for high throughput transport studies (Fig 1), we have explored thiol-ene chemistry and a novel membrane system within a microfluidic system to develop a multi-chamber microchip. Thiol-ene-based microchips may provide the opportunity for mass production of microchips [31]. Comparisons of the morphology and essential functions (e.g. tight junction formation, P-gp expression, brush border enzymatic activity) of Caco-2 cells cultured in the microfluidic device were compared with Transwell cultures to provide insights on the capability of the microchip materials for supporting tissue cell culture. To quantitatively evaluate the integrity of the Caco-2 monolayer in the microfluidic device, two pairs of electrodes that were built in-house, were embedded in the microchambers to acquire TEER. Fabricating and embedding electrodes in microfluidic devices [32–35] allowed for a simple, label-free and non-destructive technique for characterizing the barrier property of epithelial and endothelial cell layers.

## Materials and methods

### Fabrication of thiol-ene microfluidic device

Two sets of molds were required to fabricate the fluidic layers and electrode grooves of the thiol-ene microchip. The first mold was fabricated in polymethylmethacrylate (PMMA). The design of this mold was the exact replica of the thiol-ene microchip design. A second mold made of PDMS was the inverse design of the PMMA mold. The microchannel designs (200 μm (depth) x 600 μm (width)) of the top and bottom layers of the microchip were first drawn with an engineering software, Autocad (Ver 18.1). Detailed drawings with dimensions of the structures on the different thiol-ene layers can be found in S1 Fig. To fabricate the first master molds, the drawings were converted to codes by EZ-CAM (ver 15.0, Germany) and micromilled (Mini-Mill/3, Minitech Machinery Corporation, GA, USA) onto 5 mm PMMA blocks. The second molds were fabricated by mixing PDMS (DowCorning, Germany) in the ratio of 1:10 (curing agent: pre-polymer), degassed under vacuum and poured onto the PMMA master mold. The liquid PDMS was cured in the oven at 70°C for more than 20 hrs. Once the PDMS molds were cured, they were de-molded from PMMA molds, bearing the replicated design of the microchip layers.

**Fig 2:**
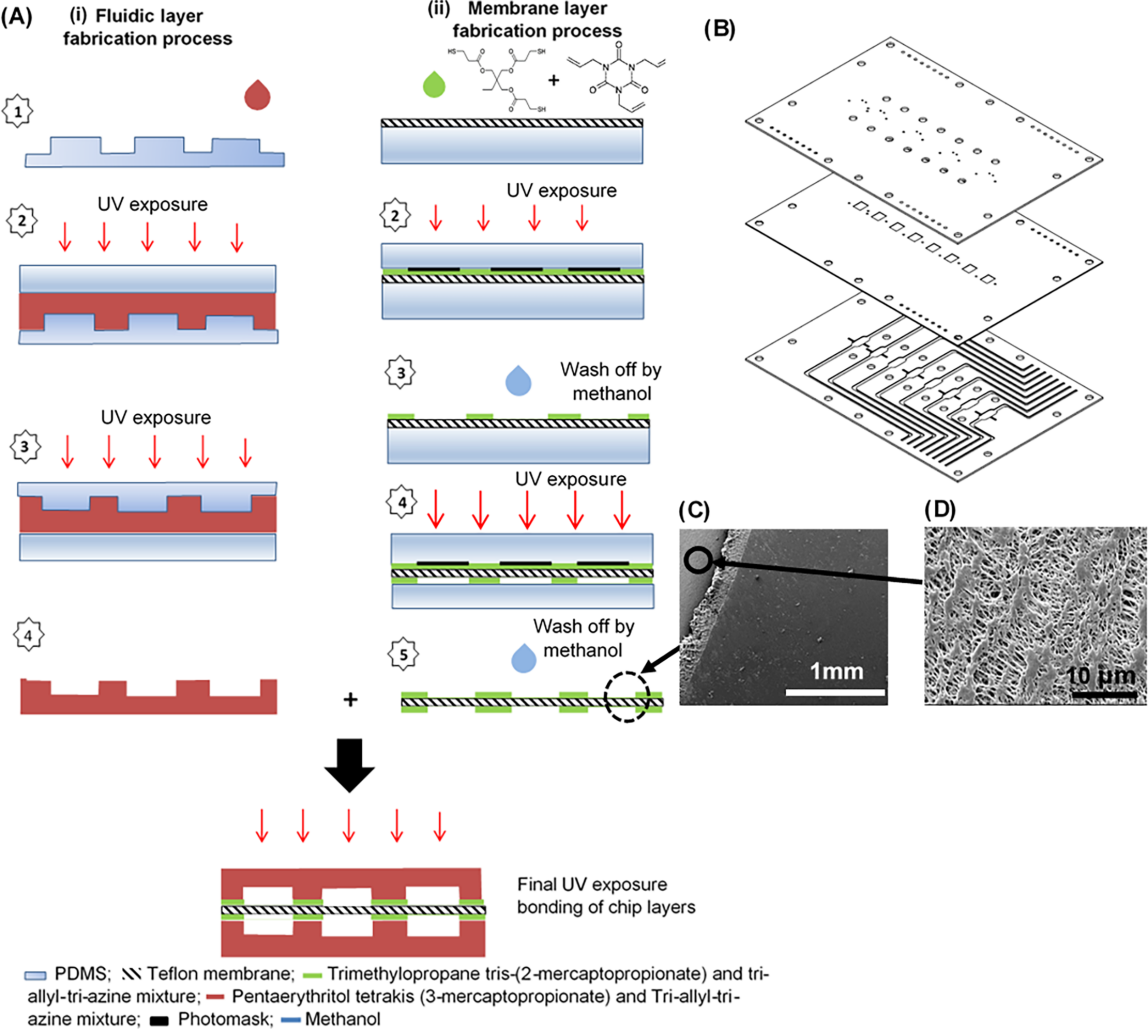
Fabrication process of the microfluidic device for Caco-2 culture. (A) Schematic process of fabricating the different layers of the thiol-ene chip (i) process of fabricating upper and lower fluidic layers; (ii) process of fabricating the thiol-ene coated Teflon membrane. (B) Exploded view of the high throughput multi-layer thiol-ene microchip for cell culture (Dimension of microchip: 76 mm x 52 mm x 2.7 mm). Thickness of the modified membrane is 0.3 mm. The membrane is coated with a thiol-ene mixture on both sides to ensure good bonding between the chip layers. Fluids were pumped in the upper and lower layers. (C) SEM images of Teflon membrane (Top view). Surface morphology was changed significantly after coating a layer of thiol-ene. The surface of the membrane has become very smooth after coating and curing a layer of thiol-ene (as indicated by red arrow). (D) Expanded view of Teflon membrane that was masked off and rinsed with methanol, maintained its porous structure in these areas.

Pentaerythritol tetrakis-(3-mercaptopropionate) (tetra-thiol moieties), 1,3,5 trilallyl-1,3,5-triazine-2,4,6(1H,3H,5H)-trione (tri-allyl moieties) and trimethylopropane tris-(2-mercaptopropionate) (3 thiol moieties) used for fabricating the fluidic layers in the thiol-ene microfluidic chips were all purchased from Sigma Aldrich, Denmark. The top and bottom layers, containing the fluidic microchannels and chambers were fabricated with a mixture of tetra-thiol moieties and tri-allyl moieties in stoichiometric ratios. The different components were mixed, poured onto the PDMS molds and exposed to UV (for 40 s on both sides (Dymax 5000-EC Series UV curing flood lamp, Dymax Corp., Torrington, CT, USA, ∼40 mW cm−2 at 365 nm) (Fig 2Ai).

To fabricate the thiol-ene coated Teflon membrane, a commercially available Teflon membrane (Millipore, Denmark) (0.4 μm in pore size; 40 μm in thickness; porosity ~ 75% according to manufacturer’s information) was modified to enable better bonding of the membrane to the thiol-ene parts containing the fluidic manifolds. A thiol-ene mixture consisting of tri-thiol moieties and the tri-allyl component as above, in stoichiometric ratios, was prepared and used to coat the membrane (Fig 2Aii). The coated membrane was exposed to UV radiation for 25 s through a plastic mask (Infinite Graphics, Singapore) that protected the cell culture regions (Step 2 in Fig 2Aii). Methanol (Sigma, Denmark) was used to rinse the entire membrane to remove any uncured thiol-ene (Step 3 in Fig 2Aii). Before bonding the layers, holes for the inlets, outlets and electrode ports were drilled through the partial cured thiol-ene layers. Thickenss of the cured Teflon membrane was measured by a profilometer (Dektak; Brunker). Next, the layers (thiol-ene coated membrane and thiol-ene fluidic manifolds) were aligned onto each other by the presence of alignment markers on the microchip layers (Fig 2A and S2 Fig). Slight pressure was applied with a roller over the layers to ensure good contact between the surfaces. To finalize the bonding, the combined layers were exposed to UV radiation for an additional minute on each side (Final step in Fig 2A).

The completed microchip would have two sets of electrodes (top and bottom) embedded into selected chambers for TEER measurements. The bottom electrodes were positioned at 1.5mm and 3mm from the entrance of the microchamber and top electrodes were positioned at 1.5mm from exit of microchamber (S2 Fig). The bottom electrodes were fabricated using an Indium alloy (InBiSn (In 51% Bi 32.5% Sn 16.5% by weight); Indium Corp, Utica, NY). Top electrodes were fabricated using Platinum wire (Pt, Advent, UK) (diameter 0.5 mm). To fabricate the bottom electrodes, the assembled microfluidic chip was first placed onto a hot plate set at 80°C for 10 min. Pieces of the InBiSn metal, with length of 5 mm, were inserted into the electrode ports (connecting to the lower fluidic layer) on the thiol-ene microchip. Slight pressure was applied manually to push the melted metal into the electrode groove. Once the electrodes were formed, electric wires of diameter 0.4 mm were inserted into the liquid metal to act as connecting wires to the multimeter (Keithley, USA). The size of electrodes was about 0.6–0.8 mm in diameter with a thickness of 0.25–0.3 mm. To fabricate the top electrodes, pieces of Pt wires (diameter of 0.5 mm, length of 5 mm) were forced fit into the electrode ports drilled through the top fluidic layer. Two connecting Cu wires were soldered to the Pt wires. To fix the electrodes in position, UV-epoxy (NOA81; Norland, USA) was applied at the junctions between connecting wires and thiol-ene chip. The entire chip was exposed to UV light for 30 s.

### Cell culture

Human Caco-2 intestinal epithelial cells used in the experiments were obtained from American Type Culture Collection ((ATCC), HTB-37, Germany). The passages of the Caco-2 cell line used in the microfluidics studies ranged from the 40^th^ to 50^th^ passages, and for the Transwell studies, passages in the range of 40^th^ to 65^th^ were used. The Caco-2 cells were cultured routinely in Dulbecco’s Modified Eagle Medium (DMEM; Sigma, Denmark). The culture medium is supplemented with 10% (v/v) heat-inactivated fetal bovine serum (FBS; Sigma, Denmark), 1% (v/v) nonessential amino acids (NEAA; Gibco, Denmark) and 1% (v/v) penicillin-streptomycin (P/S; Gibco, Denmark).

### Cell culture in Transwell inserts and microfluidic chip

#### Transwell system

Control studies were carried out using static cultures of Caco-2 cells in 12-well Transwell plates (Corning, Sigma, Denmark). The inserts in the well plate each contained a porous polycarbonate membrane (1.1 cm^2^, 0.4 μm pores). In the Transwell studies, the Caco-2 cells harvested with trypsin/ETDA solution (0.05%; Sigma, Denmark) were seeded on the top surface of the porous Transwell membrane at a density of 1 x 10^5^ cells/well. The Caco-2 cells were grown for 14–16 days in DMEM (Sigma, Denmark) supplemented with 10% (v/v) FBS, 1% (v/v) NEAA and 1% (v/v) P/S. The medium was changed every second day of cell culture.

#### Microfluidic system

After the microchip was fabricated, it was assembled onto the platform with MAINSTREAM components [36]. Briefly, the MAINSTREAM platform consists of the two peristaltic micropumps (each micropump bearing eight pumplines), cell loading components, air pressure network, glass vials acting as the reservoirs for respective inlets and outlets, two sets of LEGO Mindstorms (LEGO, Billund, Denmark) controller and motor and the PMMA platform for placing the microchip and the components (S3 Fig). The Teflon tubings (inner diameter = 0.2 mm; Bola, Denmark) micro-components and microchip were sterilized by perfusing 70% ethanol throughout the entire system at a flow rate of 5μl/min for 2 hours. Following that, sterile water was flushed into the entire system for an additional 2 hours at a flow rate of 5μl/min. It was crucial to ensure good microenvironment in the cell culture chamber and good cell attachment during cell seeding in the microchip. To achieve this, ECM known to have the ability in improving the cellular microenvironment [37–39], was used to coat the Teflon membrane. An ECM mixture of 300 μg/ml Matrigel (Corning, United Kingdom) and 50 μg/ml collagen Type I (BD Bioscience, Denmark) in serum-free DMEM was flowed into the microchip to coat the porous membrane. The ECM mixture was flowed into the microchip for 2 hours at a flow rate of 5μl/min in an incubator. After the incubation, the microchip device was perfused with cell culture medium overnight in an incubator (37°C, 5% CO_2_). Following that, the harvested Caco-2 cells from the trypsin/EDTA solution was seeded into the microfluidic chip.

For cell seeding in the microfluidic microchip, the harvested Caco-2 cells was diluted with DMEM containing 60% (vol/vol) FBS solution in order to decrease sedimentation. The re-suspended cell mixture was syringed into the ‘cell loading’ reservoirs that were assembled onto the microfluidic platform. The peristaltic micropump on the microfluidic platform was activated at a very high flow rate (flow rate ≈ 65 μl/min) to draw the cell suspension into the cell culture chambers. The microchambers were seeded with Caco-2 cells at a concentration of 2.5 x 10^5^ cells/cm^2^. A normal optical microscope was utilized to observe the distribution of the cells in the chambers. Caco-2 cells attached onto the ECM-coated Teflon membrane. Next, the entire system with the microchip was placed in the incubator (37°C, 5% CO_2_). After 2 hours, the peristaltic micropump was activated to perfuse culture medium through the upper microchannels and microchambers at a constant flow rate of 0.5 μl/min. Cell culture medium was flowed only in the upper layer on day 1 of cell culture. This was to ensure the establishment of an intact monolayer of Caco-2 cells. From the second day of cell culture onwards, the micropump that was perfusing through the lower microchannels and microchambers was also activated. Flow rate was set at 3 μl/min for both top and bottom channels. Over the 9–10 days of cell culture, phase contrast images of the cells in four different positions of each of the microchambers were taken and compared.

### Cell viability studies with thiol-ene pieces

Live/dead cell staining was conducted using LIVE/DEAD® Cell Imaging Kit (Life Technologies Corporation, UK), containing a cell-permeable dye for staining live cells at excitation/emission 488 nm/515 nm and a cell-impermeable dye for staining dead and dying cells at excitation/emission 570 nm/602 nm. Caco-2 cells were cultured in microchambers of the thiol-ene microchip for 21 days. To stain the cells, PBS was first flowed across the Caco-2 cells for about 1hr, followed by incubation with the live/dead dye solution at 37°C for 1hr. Fluorescent images of cells were taken using an inverted microscope Zeiss Axio Observer.Z1, Carl Zeiss, Germany).

The cytotoxicity studies of Caco-2 (seeding concentration of 1 x 10^4^ cells/ml) were analysed by measuring the metabolic activity of Caco-2 cells cultured in 24-well microplate using AlamarBlue® assay (Life Technologies Corporation, UK). Caco-2 cells were cultured in microwells with and without cured thiol-ene pieces. To carry out the metabolic studies, Caco-2 cells were rinsed with PBS and incubated at 37°C for 2hr with 1.5ml of culture media containing 10% of AlamarBlue® solution. Following incubation, 100μl of aliquots were placed into 96-well microplate and analysed with a microplate reader (VICTOR3™ Multilabel Counter model 1420, PerkinElmer, USA) at the excitation wavelength of 550 nm and emission wavelength of 590 nm. Wells containing medium and AlamarBlue® without cells were used as control sample. Calculation of metabolic reduction was achieved by subtracting the fluorescence values of control samples from the samples containing Caco-2 cells (with and without cured thiol-ene pieces).

### Trans-epithelial barrier measurements (TEER)

#### Transwell set-up

TEER was measured in Transwell inserts with Millicell ERS-2 chopstick Silver/SilverChloride (Ag/AgCl) electrodes (Millipore, USA), and calculated via Eq 1 (see below).

#### Microfluidic device

TEER measurements (Ω) of the Caco-2 cells cultured in the microfluidic device were obtained by coupling the connecting wires of the electrodes to a multimeter (Keithley, USA) using alligator clips. Each chamber contained two sets of top electrodes and two sets of bottom electrodes. Each time the TEER values were recorded, only one top electrode and one bottom electrode are connected to the multimeter. Four different configurations of TEER values were recorded, averaged to calculate the final TEER values measured across the microchamber.

#### TEER measurements

The final TEER values (Ω cm^2^) were determined by subtracting the baseline measurement in the absence of cells (R_baseline_) from the read out (R_1_) and multiplying with the area of cell culture (A_o,_ 1.12 cm^2^ for Transwell and 0.1 cm^2^ for the microfluidic device) as shown in Eq 1.

$$R_{1}-R_{baseline}=TEER$$ (1)

### Morphological studies of Caco-2 cells cultured in microfluidic system and Transwell cultures

Phase contrast images of the Caco-2 monolayers were obtained using an inverted microscope Zeiss Axio Observer.Z1 microscope equipped with a 10x/0.3 Plan-Neofluar objective, and a Zeiss Axiocam MRm B/W camera. (Carl Zeiss, Germany). Images were analysed with the imaging software provided by the microscope manufacturer (AxioVision 4.8.2, Carl Zeiss, Germany).

Each cell culture chamber was scanned and all images were acquired with a z-stack (6 μm between each slice) and an exposure time of 5 ms. A specific function in the imaging software, AxioVision Extended Focus module, was utilized to stitch all individual images and converted to a single image to obtain the best focused image.

Fluorescent confocal images of the monolayer morphology were obtained using a confocal microscope (Carl Zeiss, Germany). The procedure for cell staining and the dyes used were the same for both Transwell cultures and microfluidic device. Between each processing step, the cells were rinsed with PBS. The Caco-2 cells were fixed with 4% (vol/vol) paraformaldehyde (Sigma, Denmark) for 30 min, followed by 30 min of permeabilization with 0.1% Triton X-100. Next, a blocking buffer (1% bovine serum albumin (BSA; Sigma, Denmark), 0.1% Tween-20 (Sigma, Denmark) in phosphate buffer saline (PBS; Sigma, Denmark)) was introduced to the cells for 1 hr. To visualize tight junctions, immunofluorescence staining was performed using mouse anti-ZO-1 (ZO-1; Life Technologies, Denmark) diluted in the blocking buffer 1:100 and introduced into the cells and left static overnight in the fridge at 4°C. Immunofluorescence staining was also carried out to stain the mucoprotein, mucin-2. Primary mouse monoclonal antibody (ab11197; AbCam, Denmark) prepared in blocking buffer (1:100), was introduced to the cells. The samples were protected from light and left static overnight in the fridge at 4°C. Next, the cells were rinsed with PBS, followed by counter-stained with the secondary antibody (AlexaFluor 488 goat anti-mouse; Life technologies, Denmark) prepared in blocking buffer (1:200) and left static in room temperature for 2 hrs. Similar immunofluorescence staining procedure was carried out to stain the P-gp transporters. Primary rabbit polyclonal antibody (ab129450; Abcam, Denmark) prepared in blocking buffer (1:200) was introduced to the cells and left static overnight in the fridge at 4°C. Following that, the cells were rinsed with PBS and stained with secondary antibody (AlexaFluor 488 goat anti-rabbit IgG H & L; Abcam, Denmark) prepared in blocking buffer (1:200). The cells were left in static condition in room temperature for 2 hrs. Staining of the nucleus and actin were carried out by diluting 7-aminoactinomycin D (7-AAD; Invitrogen, Denmark) (32 μM) and Rhodamine phallodin (RP; Life Technologies, Denmark)) to 1:100 in PBS and incubated with the cells for 1 hr. Lastly, mounting media (Vectashield; VWR, Denmark) was added to the cells to protect the fluorescent dyes. For the Transwell cultures, the membranes were removed from the inserts and mounted onto glass slides before microscopic imaging. Staining of cells in the microfluidic device, were performed *in situ* within the microchannels by flowing the different reagents into the microchannels and microchambers via MAINSTREAM platform. Visualisation of the tight junctions, mucus and P-gp transporters were carried out at excitation/emission wavelength of 488/570 nm. The stained nuclei were visualised at excitation/emission wavelengths of 546/647 nm. Fluorescent imaging of the stained Caco-2 cells on the thiol-ene microchip was performed through the thiol-ene layer on an upright microscope (ZEISS Axioscope; Carl Zeiss, Germany). Similar to the phase contrast images, each cell culture chamber was scanned and all images were acquired with a z-stack (6 μm between each slice). The recorded images of the cells were analyzed with an imaging process software ImageJ.

### Aminopeptidase studies to identify differentiated Caco-2 cells

L-alanine-4-nitroaniline hydrochloride (L-4AN; Sigma, Denmark) was prepared by dissolving the L-A4N substrate in DMEM without phenol red (DMEM^-PR^, Gibco, Denmark) to a concentration of 1.5 mM. In the Transwell studies, the Caco-2 cells were first rinsed with DMEM^-PR^ in both the apical and basolateral sides for 3 times. 500 μl of L-A4N substrate solution was added to the apical side of the cells and 1500 μl of DMEM^-PR^ was added to the basal lateral side of the cells and incubated at 37°C. Sample aliquots of 100 μl was removed from the apical side at 30 min intervals and transferred to a 96-well microplate. Studies were carried out for a 2 hr period. Analysis of the sample aliquots were carried out with a microplate reader (Victor 3V; Perkin Elmer). DMEM^-PR^ was set as the reference. The test was calibrated with a series of dilutions of 4-nitroaniline in DMEM^-PR^. One unit is defined as the hydrolysis of 1.0 μmol of 4-nitroaniline per minute. All of the reagents preparation and experimental studies were conducted under the protection of light. The aminopeptidase experiments in the Transwell cultures were carried out on *in vitro* cell culture day 5 and 21.

In the microfluidic device, the aminopeptidase studies were carried out on day 5 of *in vitro* cell culture. DMEM^-PR^ was first perfused to both the top and bottom fluidic channels for 45 min at a flow rate of 3 μl/min. Next, 1.5 mM of L-A4N solution was flowed into the upper microchannels and microchambers of the thiol-ene microchip at a flow rate of 3 μl/min. Sample aliquots of 120 μl were removed from the outlets of the upper microchannels at every 30 min and transferred to a 96-well microplate. Similar to the Transwell studies, the sample aliquots from the microfluidic system were analyzed for the cleaved product, 4-nitroanalide with the microplate reader.

### Permeability and efflux studies in cell monolayers

The compounds used in the permeability studies, [^3^H]-mannitol (PerkinElmer; USA), fluorescein isothiocyanate (FITC)-labeled dextran (FD4; 4kDa; Sigma, Denmark), insulin (Novo Nordisk, Denmark) and tetradecyl-β-D-Maltoside (TDM; Sigma, Denmark) were all prepared using buffer^+^ as the diluent. Buffer^+^ was prepared by mixing Hank’s Buffered Saline solution (HBSS; Gibco, Denmark), 0.1% (wt/vol) OVA (ovalbumin; from chicken egg white, Sigma, Denmark) and 10 mM HEPES (HEPES; Sigma, Denmark) at pH 7.4. The different compounds were prepared in various concentrations: 0.8 μCi/ml [3H]-mannitol, 100 μM insulin (peptide/drug) or 540 μM FD4, and 0 or 400 μM TDM.

In the Transwell studies, before carrying out the transport experiments, DMEM was changed to buffer^+^. 400 μl of buffer^+^ was added to the apical side and 1 ml to the basolateral prior to equilibrate the Transwell plate for 60 minutes. Buffer^+^ was replaced with 400 μl test solution at the apical side at time zero, and the Transwell plates were incubated at 37°C and 5% CO_2_ with gentle shaking. The gentle shaking was to ensure there was little unstirred diffusion layers of fluid in the basolateral region. Basolateral samples were collected every 15 minutes for 1 hour and analyzed along with apical test solutions for [^3^H]-mannitol content in a scintillation counter (Packard TopCount; PerkinElmer), after mixing with scintillation fluid (Microscint-40; PerkinElmer), along with a peptide and FD4 content. After the experiments, the cells were washed twice with buffer^+^ and replenished with medium for 24 hour recovery. In the microfluidic system, similar to the Transwell studies, before the start of experiment, the DMEM was replaced with buffer^+^. Buffer^+^ was flowed into the system for 1 hr. Subsequently, the buffer was changed to the test solutions in the top fluidic layer. Flow rate was set at 3 μl/min (in both the upper and lower layers). In the collection reservoirs of the basolateral chambers, 200 μl of buffer was added to each of the collection reservoirs. This would enable sample aliquots of 100 μl collected at every 15 min intervals. Dilution by perfusion was factored in in the calculation of the permeability of the compounds across the cell monolayers.

Before carrying out the Rh 123 studies, buffer^+^ was flowed to both the upper and lower channels for 1 hr. Following that Rh 123 (Sigma-Aldrich, Denmark) prepared in buffer^+^ to a concentration of 10 μM was flowed to the basolateral side. 200μl of buffer^+^ was added to each waste reservoirs. At each 15min interval, 150 μl was removed from the respective waste reservoirs with replacement of 150μl of buffer^+^. Studies were carried out in triplicates.

### Data analysis of permeability results

The Caco-2 translocation of peptide, [^3^H]mannitol or Rh 123 over Caco-2 layers is expressed as the apparent permeability (P_app_), given by:

$$P_{app}=\frac{dQ}{dt}\frac{1}{A\;C_{0}}$$ (2)

Where dQ/dt is the steady-state flux across the cell layer (pmol/s), A is the surface area (1.12 cm^2^ for Transwell, 0.1 cm^2^ for microfluidic), and C_0_ is the initial sample concentration [8].

The basolateral samples were analyzed for insulin content using commercial insulin enzyme immunoassay kit (EIA, Phoneix Pharmaceuticals, Germany). Standards were prepared from test solutions, and were fitted to Eq 3 using Prism-6 (GraphPad, Ver 6).

$$Abs(450nm)=A+\frac{B-A}{1+10^{\left((log{EC}_{50}-x)C\right)}}$$ (3)

where *x* is log(concentration) of peptide in M, and A, B, C and EC_50_ are fitting parameters [40].

Basolateral samples were analyzed for FD4 content in a fluorescence plate reader (MD Spectramax Gemini, USA) with excitation/emission of 490/525 nm, based on standard curves prepared from test solutions. Statistical analysis was carried out using the software Prism-6, where unpaired Students t-tests were used for comparison, and a significant difference was considered if p < 0.05. Results are presented as the mean ± standard deviation of the mean (SEM).

For the P-gp studies, the collected samples were analyzed for Rh 123 content using a fluorescence plate reader with excitation/emission of 485/546nm, based on standard curves prepared from test solutions. The efflux ratio of P-gp was determined by:

$$efflux\;ratio=\frac{P_{app\;(Basal\to Apical)}}{P_{app\;(Apical\to Basal)}}$$ (4)

Similar to the permeability studies, all P-gp results were calculated as mean ± standard error (SEM). Statistical evaluation of the quantified data was carried out using the software, Prism-6. A significant difference was considered when p < 0.05.

## Results and discussion

### Development of thiol-ene-based microchip for Caco-2 cell culture

We designed and developed a thiol-ene based microchip that consists of eight cell culture micro-chambers where each cell culture chamber has two compartments (to become the apical and basal side of the cell layer, respectively) (Fig 1). The developed microchip contains eight micro-chambers, thus allowing parallel culturing of Caco-2 cells or other tissue models (Fig 2B). The design of eight channels allows for significant controls during drug transport studies and allowed us to employ the previously reported microfluidic flow system developed by others [36]. Thiol-ene was chosen since thiol-ene polymers have been reported to have low volume shrinkage [41,42] and are biocompatible for use in cell culture [43]. Additionally, thiol-ene polymers have a low affinity to absorb molecules [41,42,44]. For instance, an earlier study showed that when Rhodamine B was incubated in microchannels fabricated from thiol-ene polymers and PDMS, respectively, increased fluorescence was observed on the outside of the PDMS channels as compared to the thiol-ene channels [44]. The thiol-ene-based microchip has an external dimension of 76 mm x 52 mm x 2.7 mm and consists of three layers. The detailed dimensions of the structures on the different chip layers are shown in S1 Fig. The top and bottom layers of the microchip were fabricated via the method reported by Lafleur et. al. [45] using a two-step UV exposure [46,47]. In this method, curing of the thiol-ene polymers was carried out without a photoinitiator present; it should be noted that this will typically not lead to curing with regular UV-light sources [41,42,45–48]. Extra care was taken during the UV-light exposure of thiol-ene polymers to avoid complete curing of stoichiometric thiol-ene [46], since this will prevent strong bond formation between the different thiol-ene layers during the bonding step.

The porous Teflon membrane—sandwiched between the thiol-ene fluidic layers—became transparent to visible light when wetted (S4 Fig), thus allowing real-time and fluorescence microscopic monitoring of the Caco-2 cells cultured on it. The bonding of the thiol-ene top and bottom fluidic layers with the Teflon membrane required a dedicated modification of the membrane. The porous Teflon membrane was coated with a thiol-ene mixture and exposed to UV radiation with a plastic mask that protected the part of the membrane to be used for cell cultures. Methanol was used to rinse the entire membrane to remove any traces of uncured thiol-ene. The end result was a thiol-ene modified membrane with regions, which were not coated with thiol-ene and thus allowed the porous Teflon membrane to be used for cell culturing (S3 Fig). Thickness of the regions that were not coated with thiol-ene remained as 40μm, while regions that were coated with cured thiol-ene was 300μm in thickness. When examined with a scanning electron microscope (SEM), a smooth surface was observed in regions where the porous Teflon membrane was coated with thiol-ene and exposed to UV light (Fig 2C). In regions that were masked and rinsed with methanol, the porous structure of the Teflon membrane was preserved (Fig 2D). This procedure clearly demonstrated that thiol-ene ‘click’ chemistry can be exploited to functionalize and pattern the membrane surface [46,47]. In the pressure burst studies of the microfluidic chip (S5 Fig), the multi-layer microchip could withstand burst pressures of more than 6 bars (S1 Table). The presented method of fabricating the microfluidic chip can easily be carried out at room temperature and in standard laboratory environments, therefore eliminating the need for costly or specialized cleanroom facilities.

### Biocompatibility of the thiol-ene material

**Fig 3:**
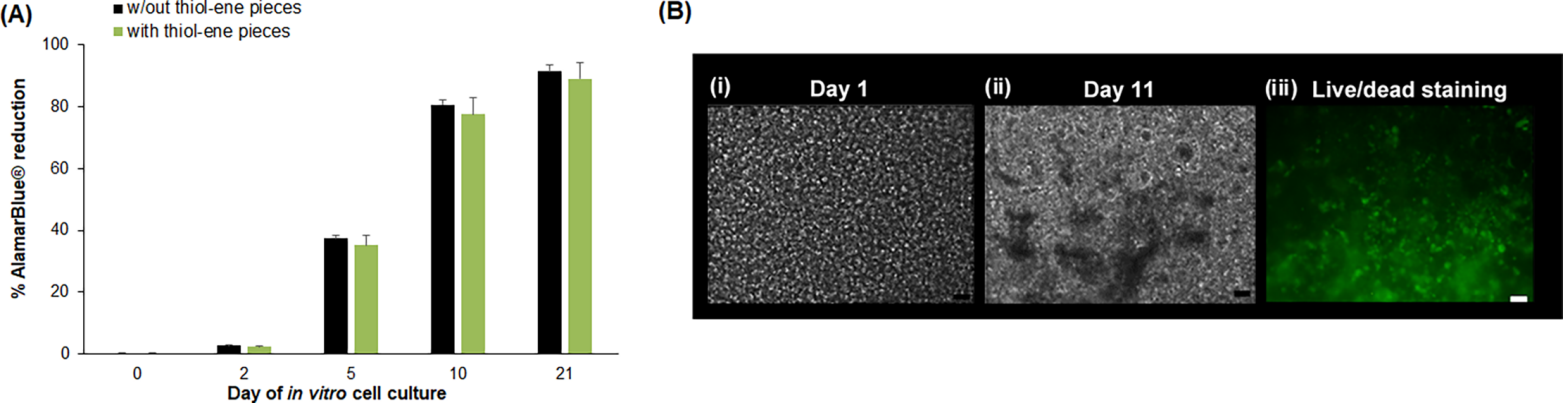
Biocompatibility of thiol-ene. (A) Metabolic activity of Caco-2 cells (AlamarBlue® assay) over day 0 to day 21 of *in vitro* cell culture. (n = 3; mean ± SEM; scale bar = 50μm) (B) Microscopic images of Caco-2 cells cultured in thiol-ene microchip (i) Phase contrast image of Caco-2 cells cultured on day 1 of *in vitro* cell culture; (ii) Day 11 of *in vitro* cell culture; (iii) live/dead cell staining of Caco-2 cells on day 11 of *in vitro* cell culture.

Caco-2 cells were cultured with and without pieces of cured thiol-ene material in microtiter plate wells. The metabolic activity was assessed using AlamarBlue® assay for selected days during the culture (Fig 3A). The percentage reduction of AlamarBlue® for cell cultures with thiol-ene pieces were comparable to microwells with no thiol-ene pieces, indicating that the thiol-ene had no adverse effect on the metabolic activity. The metabolic activity of the Caco-2 cells increased steadily from days 0 to 21 of *in vitro* culture. For both control cultures and cultures in the presence of thiol-ene pieces, the rate of metabolic activity of Caco-2 cells were higher between day 0 to 5 of *in vitro* culture compared to day 5 to 10 and day 10 to 21 respectively. The metabolic activity is likely related to the proliferation of Caco-2 cells. In the literature, it was shown that proliferation of Caco-2 cells takes place after 48hr of seeding the cells and proliferation rate of Caco-2 is most rapid between day 3 to day 9 of cell culture [10,49], which is consistent with our results. At day 21, the metabolic rate is not much higher than at day 10 which may indicate that once the proliferation has stopped (around day 10), the metabolic activity showed only modest increment during differentiation. An earlier study showed comparable biocompatibility of three different cell lines cultured on standard cell substrates and thiol-ene material [50]. This further emphasized the biocompatibility of thiol-ene as a suitable cell culture material.

**Fig 4:**
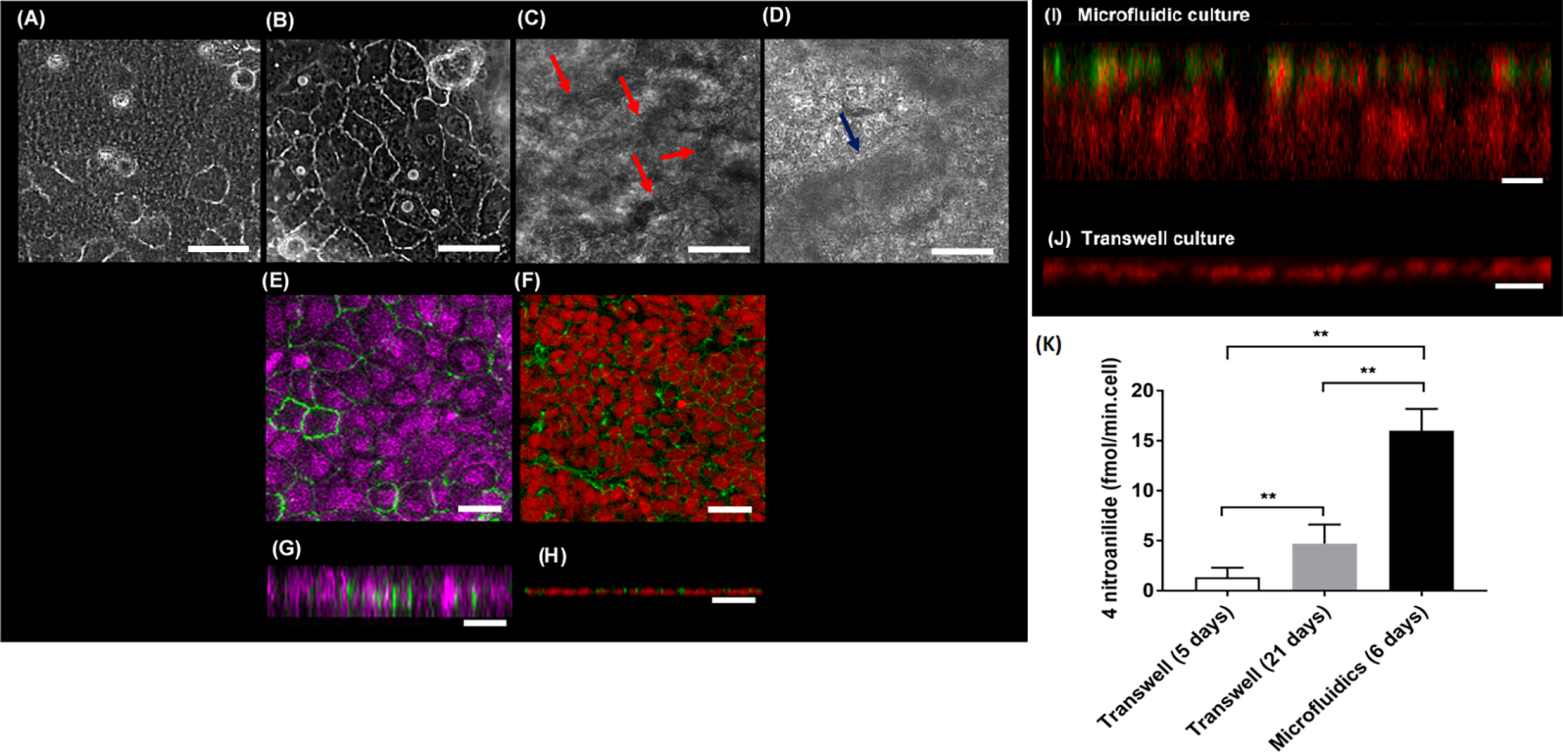
Morphology of Caco-2 cells cultured in the microfluidic device. Caco-2 cells cultured in microfluidic system (A-G). Caco-2 cells cultured in Transwell system (H-I). Phase contrast images of the Caco-2 cells cultured in the microchambers on the thiol-ene microchip over 10 days. (A) Day 1; (B) Day 2; (C) Day 5; (D) Day 8. Cells multiply and differentiate over the days of culture. Folds in the monolayer of Caco-2 cells start appearing from day 4 of cell culture. The folds in the Caco-2 monolayers are more prominent from day 5 onwards (indicated by red arrows). Dark ‘patches’ also start appearing on the Caco-2 monolayers from day 7 onwards. They become more prominent from day 8 of cell culture (indicated by blue arrow). (E) Immunostaining of zonula occludens-1 (ZO-1) (green) and nuclei (magenta) for Caco-2 cells cultured in microfluidic device. (F) Caco-2 cells cultured in Transwell stained for nucleus and ZO-1 (Nuclei in red and ZO-1 in green) (Day 21). (G) Vertical cross-section view of the Caco-2 monolayer (nuclei in magenta, ZO-1 in green). The Caco-2 cells are ≈ 40 μm– 50 μm in height on day 3 of cell culture in the thiol-ene microfluidic chip. (H) Vertical confocal image of Caco-2 cells in Transwell (Nuclei in red and tight junctions in green). Immunofluorescence staining of nucleus and mucus on Caco-2 cells cultured in: (I) Thiol-ene microchip on day 10 of cell culture (nucleus in red, mucoprotein 2 (MUC-2) in green). The fluorescent images of the cells demonstrate that the cells have polarised into columnar cells of about 100 μm in height and formed villous-like structures. (J) Cells in the Transwell inserts were stained for nucleus and mucoprotein 2 at day 21. Only the nuclei could be fluorescently imaged but not MUC-2. Height of cells were about 25–30 μm at day 21. Cells were photographed at 10 x magnification. (Scale bar = 50 μm) (K) Differentiation of Caco-2 cells cultured in Transwell inserts and thiol-ene microchip as indicated by the activity of the brush border enzyme aminopeptidase. (n = 3, mean ± SEM; * p < 0.03, *** p < 0.001, **** p < 0.0001).

The culture condition on chip under perfusion is however very different compared to batch cultures for assessing biocompatibility. Caco-2 cells were seeded on the chip and cultured under flow condition. Phase contrast images of the Caco-2 cells cultured in the thiol-ene microchip were taken after seeding and over the days of culture. Cells adhered well on ECM modified membrane (S6 Fig) and less well on unmodified membrane (S7 Fig). ECM seem critical, as very few cells were observed on unmodified membrane after 5 days of cell culture under perfusion (Compare figures S7B Fig, Fig 3B and Fig 4). The apparent dependence on ECM for culturing Caco-2 cells is supported extensively in the literature [37–39,51–54]. At day 1 of *in vitro* cell culture (after 24hr of continuous perfusion of cell culture media across the cells), at least 80% of all the microchambers showed Caco-2 coverage (Fig 3Bi). From day 1 to day 11 of *in vitro* cell culture, Caco-2 cells in the microchambers were observed to proliferate and differentiation of cells took place at day 6 of *in vitro* culture (results not shown). At day 11 of *in vitro* cell culture, ‘dark patches’ (indicated by red arrows in Fig 3Bii) were observed on the cells. At day 11 of *in vitro* cell culture, the live/dead staining showed that > 95% of the Caco-2 cells cultured in all the microchambers on the thiol-ene microchip were alive (Fig 3Biii) indicating excellent biocompatibility of the thiol-ene material.

### Characterization of Caco-2 cell growth pattern under flow conditions

The characterization of Caco-2 growth pattern was under the following conditions: cell culture medium was perfused through the upper and lower fluidic layers at a flow rate of 3 μl/min for 9–10 days of *in vitro* cell culture. A flow rate of 3 μl/min corresponds to a dynamic shear stress of 0.008 dyn/cm^2^, which falls within the range of what cells experience in the human intestines [55,56]. Caco-2 cells were observed to adhere onto the ECM coated Teflon membrane within 30 min after cell seeding. During the phase of active growth, it was observed that once the flow of cell culture media was started (first 24 hrs of cell culture), the Caco-2 cells spread out [57], although, in some regions the cells grew in clusters (Fig 4A). It was also observed that flowing DMEM at 0.5μl/ml for the first 16-24hrs across the cells, was sufficient to wash-out the unattached Caco-2 cells (S6B Fig) without releasing the cells. By day 2 (Fig 4B), the cells had distinct polygonal shapes with clear, sharp boundaries between the cells. The shape and morphology of the Caco-2 cells in the microfluidic device were similar to the cells cultured in a microplate (results not shown). Confluent monolayers of Caco-2 cells were observed in the microfluidic device typically around day 3–5 of cell culture. From day 5, it was apparent that cells grew on top of each other suggesting the formation of folds (4B and C) and it became more challenging to observe the cells through an optical microscope (Fig 4C). It is unclear if the folds indicate the formation of villi structures. From day 7 onwards, the appearance of ‘dark’ patches (Fig 4D) were observed on the cells.

The establishment of apical tight junctions determines the integrity of the human intestinal epithelial cell monolayer [8]. To visualize and validate the presence of apical tight junctions in the Caco-2 cells cultured in the microfluidic device, immunofluorescence staining using antibodies directed at the tight-junction protein, ZO-1, was carried out.

The immunofluorescence images confirmed the formation of confluent Caco-2 monolayers, expressing tight junctions (Fig 4E and 4F). Analysis of the vertical sections of the confocal images of the Caco-2 cells (Fig 4G) revealed that the tight-junction proteins were situated between neighbouring cells at the apical side of the Caco-2 cells (Fig 4E and 4G). Images of the cells cultured in the Transwell inserts at day 21 appeared cuboidal with a measured heights of 14–20 μm (Fig 4H). However, the Caco-2 cells cultured in the thiol-ene microfluidic device (three days of cell culture) were observed to appear columnar in shape with heights of ≈ 40–50 μm according to measurements with confocal microscopy (Fig 4G). The Caco-2 cell layer grew in height after prolonged culture (Fig 4G) while corresponding Transwell plates cultures remained thin (Fig 4H). It is unclear if the growth in height is due to Caco-2 cells growing on top of each other or if the cells are elongated. The Caco-2 cells cultured in our microfluidic device is about the same columnar size and shape (40–50 μm) as reported in healthy human intestinal epithelial cells [58]. We can confirm previous results indicating that perfusion [17] seem to stimulate the Caco-2 cells to polarise into columnar cells that were almost 2 fold taller than the cells from Transwell inserts.

In the reported microfluidic device, as the cell culture period progressed in the microfluidic device, from microscopic phase contrast images, there were observable regions of ‘dark’ patches (Fig 4D, S8 Fig). These ‘dark’ patches started appearing from day 7 of cell culture but became more prominent from day 8 onwards. We observed that these dark patches were absent when the cells were cultured at a low flow rate of 0.5 μl/min (results not shown). To further investigate the nature or origin of these ‘dark’ patches, the Caco-2 monolayers were stained for the muco-protein, Mucin-2 (Mucin-2 is commonly found in human intestines) on day 10 of cell culture. Immunofluorescence staining directed towards the protein Mucin-2 showed positive stains at the apical surfaces of the villous Caco-2 monolayers (Fig 4I). Mucus production was not observed in static Transwell cultures (Fig 4J) corroborating previous findings [19], but has previously been reported for microfluidic devices [14,17,59]. The mucus production may stem from the gastrointestinal tract’s defence mechanism towards mechanical stress [60–62]. Although Caco-2 may have derived from an adenocarcinoma, under desirable conditions (e.g. stromal factors [63], mechanical cues [17,59]), Caco-2 cells may lose control in differentiating to exhibit capabilities and morphologies that are otherwise not commonly seen. For example, a study reported on embedding Caco-2 cells into a collagen matrix and culturing them *in vivo*, resulted in cells expressing either mucous, enteroendocrine, Paneth or absorptive cell markers [63]. Thus, suggesting that Caco-2 cells may contain different lineage progenitors or they are multipotential. Previous reports have linked the mucus production to higher fluid flow and cyclic peristaltic motions [14,17,59]. Although the fluidic shear is lower here (≈ 0.008 dyn/cm2 at 3μl/min), it appears to cause production of Mucin-2, whereas Transwell cultures did not show the presence of Mucin-2 on the Caco-2 monolayers (Fig 4J).

Differentiated Caco-2 cells express intestinal brush border enzymes [10,12], so to further evaluate the differentiation of microchip Caco-2 cells compared to Transwell cultures, we measured the level of aminopeptidase activity via conversion of the substrate L-alanine-4-nitroaniline hydrochloride (L-A4N). Both systems were tested on day 5 of culture, whereas Transwell monolayers were additionally tested on day 21. In Transwell cultures aminopeptidase activity increased by more than 5-fold between day 5 and day 21, consistent with earlier findings [12,17]. In microfluidic device cultures, at day 5, the aminopeptidase activity was higher than in the Transwell cultures at day 21 (Fig 4K), consistent with earlier findings in microfluidic devices [14,17]. These results indicated that Caco-2 cells cultured in the presence of continuous flow required a shorter time to polarize and differentiate.

### Barrier functions

**Fig 5:**
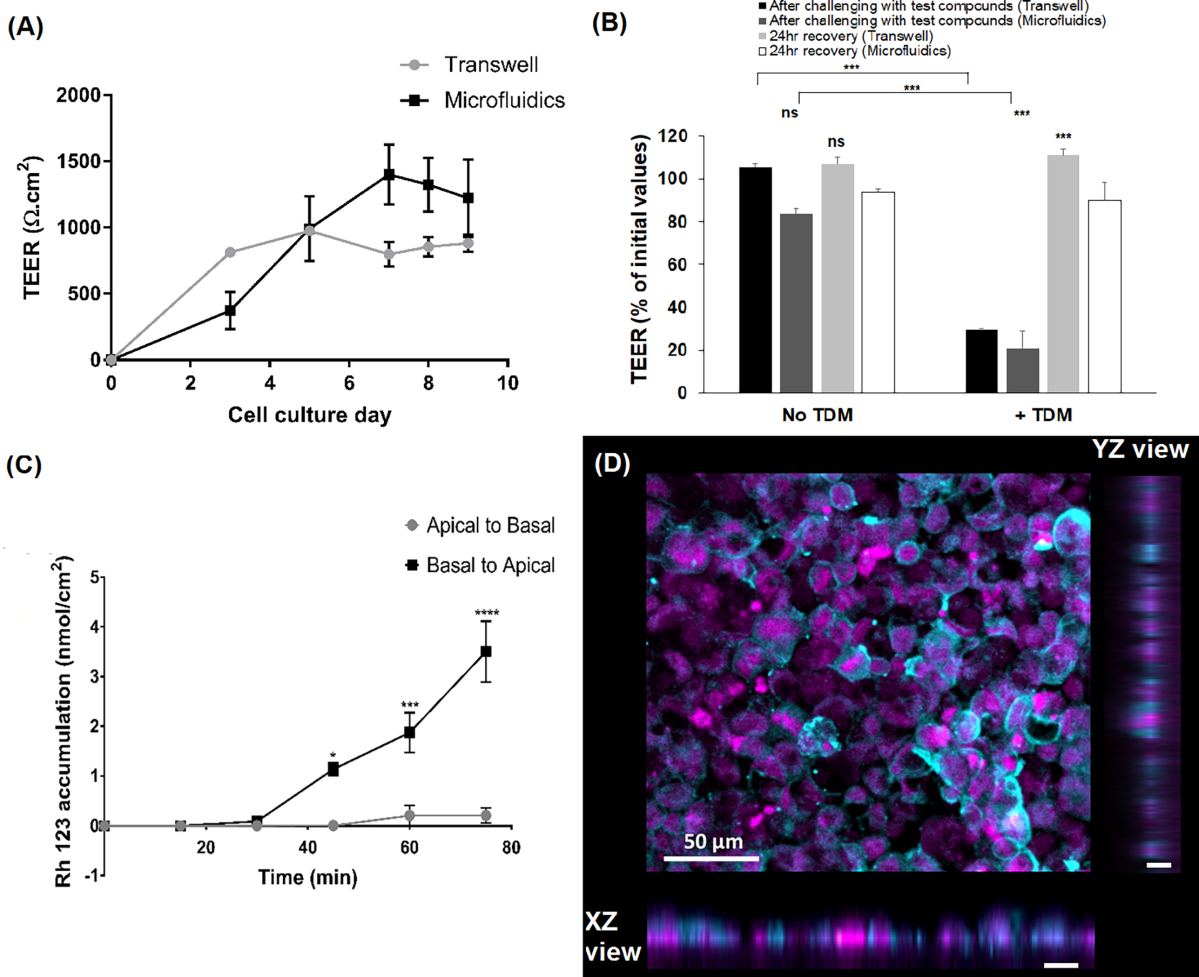
Barrier functions of Caco-2 cell monolayers in the microfluidic device or Transwell system. (A) TEER measurements of Caco-2 cells cultured in thiol-ene microchip and Transwell inserts for the same cell concentration of 2.55 x 10^5^ cells/cm^2^. (Here, number of measurements per data point n = 12 for microfluidic device; and n = 5 for Transwell inserts). (B) Effect of test compounds alone or with TDM on Caco-2 TEER in the Transwell or microfluidic system, immediately after the experiment or following 24 h recovery in medium. (C) Rh 123 accumulation profile in the basolateral and apical chambers across Caco-2 monolayers in microfluidic device. Data points represent mean ± SEM (n = 3). Where ns = not significant and *** p < 0.001. (D) Immunofluorescence staining of P-gp on Caco-2 cells cultured in microfluidic device (nucleus in magenta, P-gp in cyan). Magnification 20x; scale bar = 50 μm.

TEER is a widely used technique for measuring epithelial cell monolayer tightness and integrity. To approach an equal potential drop over the entire membrane [64], the electrodes in the microfluidic device were embedded directly above and below the membrane. A low-melting indium alloy (InBiSn) was used for the bottom electrodes and platinum wires for the top electrodes. TEER measurements were obtained by coupling the electrodes to a multi-meter (Keithley, USA) that supplied DC signals (constant current = 10 μA). Dissimilar materials were chosen for fabricating the electrodes due to the properties of the metals. InBiSn is a very soft and brittle (Brinell hardness = 11 HB [65]) metal. To place the InBiSn metal as top electrodes this will require heating of the microchip to soften the microchip layers to insert the InBiSn metal. Hence, melted metal may drip onto the porous membrane in the microchamber. Alternatively, by force-fitting the metal into the electrode ports, would have resulted in breakage of the metal. Due to these challenges, platinum was chosen for the top electrodes. TEER measurements of the Caco-2 monolayer cultured in the thiol-ene microchip were taken from day 3 onwards of cell culture and monitored over the remaining days of the experiments. Importantly, the TEER measurements of the Caco-2 cells cultured in the thiol-ene microchip and the transwell plates showed a significant increase over the first days of recording (Fig 5A). Both culture systems reached a plateau phase in terms of TEER. The TEER values from the presented microfluidic device displayed larger variation compared to the Transwell setup with commercial TEER measurement equipment, which may be due slight differences in electrode design and position in the 8 microchambers [66], and may be optimized in future device generations.

To validate the accuracy and dynamic span of TEER values acquired from the electrodes on the microfluidic chip, a permeation enhancer, tetradecyl-β-D-maltoside (TDM) [67–69], was introduced to the Caco-2 monolayers for two hours. The concentration of TDM used in the earlier reported studies is expected to cause a transient disruption of the Caco-2 monolayer integrity, while causing minimal cell death [67,68], which is reversible after 24 hours in fresh cell culture medium. In the presence of TDM, the TEER values measured in the microfluidic device dropped to 21% of their initial values, and recovered to 90% after 24 hours of continuous perfusion with cell culture medium, consistent with results from static Transwell cultures (Fig 5B). This indicates that the electrodes fabricated on the microfluidic device were sensitive to detect dynamic changes in the integrity of Caco-2 monolayers.

Another important characteristic present in differentiated Caco-2 cells is the presence of efflux transporter P-gp. These P-gp transporters play an important role in determining the bioavailability of drugs, especially the orally administered drugs. We evaluated the presence of P-gp transporters in the Caco-2 monolayers cultured in the microfluidic system by carrying out permeability studies with a well characterized substrate, Rh 123 (as a model drug for uptake and transport) on day 10 of cell culture (Fig 5C). Rh 123 was flowed through either the apical or basal compartments and sample aliquots were collected accordingly from the basal or apical collection reservoirs. The apparent permeability in the apical-to-basal direction was P_app(apical → basal)_ ≈ 1.03 x 10^−6^ cm/s. However, P_app(basal → apical)_ ≈ 1.12 x 10^−5^ cm/s, suggesting that Rh 123 was transported across the Caco-2 monolayer by the P-gp transporters in the basal to apical direction. The resulting efflux ratio ≈ 10.8 was comparable to previously reported values for static Transwell cultures [70–72]. An efflux ratio of higher than 2 is a strong indicator that P-gp transporters are present in the Caco-2 cells [73]. To further confirm the presence of P-gp transporters in the Caco-2 monolayers, immunofluorescence staining towards the P-gp transporters was performed (Fig 5D).

### Permeability studies of FITC–dextran (FD-4), mannitol and insulin in the presence or absence of permeability enhancer

**Fig 6:**
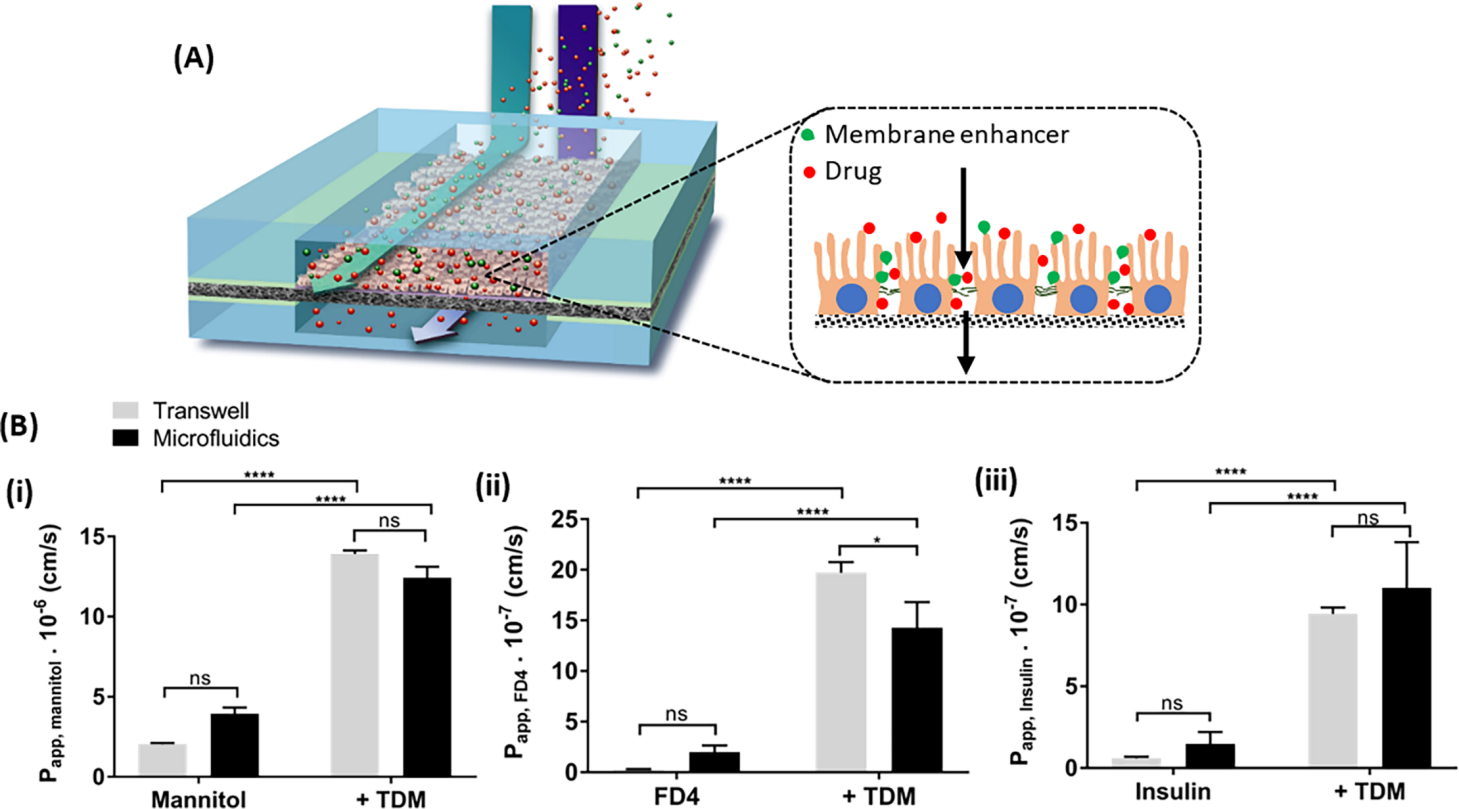
Permeability studies of Caco-2 layers with different compounds. (A) Schematic drawing of membrane enhancer and drugs flowed across the Caco-2 cells cultured in the microfluidic device. Insert is an enlarged schematic view of disrupted tight junctions upon co-administering the membrane enhancer TDM. (B) Comparison of permeability profiles of different compounds with or without TDM experimented on Caco-2 layers cultured in microfluidic device versus Transwell: (i) mannitol, (ii) FD4 and (iii) insulin. Data points represent mean ± SEM (n = 4; *p ≤ 0.02, **** p < 0.0001 and ns = not significant).

To further assess the rate-limiting barrier of the Caco-2 monolayers cultured in the thiol-ene microchip, the behaviour of three different test compounds (mannitol, fluorescein isothiocyanate (FITC)-labelled-dextran (FD4) and insulin) was studied (Fig 6A). Mannitol and dextran were used as model drugs. Insulin was used due to the interest in oral delivery of this drug in the pharmaceutical industry to provide a proof of concept of the Caco-2 monolayers cultured in the thiol-ene microchip for drug permeability studies. Insulin is usually administered by the subcutaneous route [74,75]; however, orally delivered insulin is attracting considerable attention due to the improved compliance this would provide for diabetic patients [74]. Oral delivery of insulin has major limitations, due to the degradation of insulin by proteolytic enzymes in the gastrointestinal tract and poor intestinal barrier permeability due to its molecular weight [75]. The oral bioavailability of insulin can be improved by e.g. co-administration of permeability enhancers [67–69,76,77].

The permeabilities (P_app_) for mannitol, FD-4 and insulin were slightly higher (not significant) in the microfluidic chip system at day 9 or 10 of cell culture, compared to the static Transwell cultures at day 14 of *in vitro* cell culture (Fig 6B). To further investigate whether the permeability of different test compounds was affected by the introduction of the permeability enhancer TDM, Caco-2 monolayers in microfluidic device or Transwell inserts were exposed to mixtures of test compounds with and without TDM (Fig 6A). In both systems, the P_app_ values for all three compounds greatly increased in the presence of TDM, with significant differences (p < 0.0001) between the pre- and post TDM studies for both cell culture systems (Fig 6B). The results demonstrated that the addition of TDM permeabilized the Caco-2 monolayers in both systems and allowed substantial transport of compounds across the Caco-2 monolayers. Additionally, the P_app_ values of the test compounds in the presence of TDM were comparable between the static Transwell cultures and the microfluidic system (p < 0.0001 for mannitol and insulin studies but p < 0.02 for FD4 studies). In both systems TEER was reversibly decreased following TDM addition, recovering to normal levels within 24h in fresh medium (Fig 5B). The continuous flow of culture media across the cells in the microfluidic device may have aided the recovery of barrier property, although to our knowledge, the time-course of Caco-2 monolayer recovery following absorption enhancer challenge in microfluidic devices has not previously been reported. This phenomenon observed in the Caco-2 monolayers cultured in the microfluidic device further strengthen the potential of such *in vitro* platforms for ADME-Tox studies.

## Conclusion

A thiol-ene based multi-chamber and multi-layer microfluidic chip was engineered to provide a controlled platform to sustain long-term Caco-2 cell cultures under fluidic flow for transport studies. Characterization of the microfluidic chip revealed that the functionality of the porous Teflon membrane (sandwiched between the top and bottom fluidic layers) could be changed by coating and curing it with a thiol-ene mixture, followed by ECM coating of the porous region of the Teflon. Thus, bonding the Teflon membrane between two cured thiol-ene layers within a fluidic system formed a microchip that could support long time cell culture. Growth and differentiation of Caco-2 cells cultured in the thiol-ene microfluidic chip accelerated under continuous flow conditions. The Caco-2 cell monolayers formed a tight barrier with P-gp transporters and mucus production, effectively mimicking the human intestine and serving as a functional drug transport model. The performance in terms of barrier function of the chips was very similar to Transwell plates. The described chip may therefore be a suitable brick in a human-on-a-chip concept.

## Supporting information

S1 Fig Cad drawings of the different layers in the thiol-ene microchip. (DOCX)

S2 Fig Schematic overview of thiol-ene microchip with electrode ports and alignment markers. (A) Schematic drawing of the microfluidic layers stacked together. (B) Enlarged view of the microchamber with the electrode ports.
(DOCX)

S3 Fig Assembled thiol-ene microfluidic chip with the cell culture platform. Blue arrow indicating the micropump perfusing through the top layer. Red arrow indicating the peristaltic micropump perfusing through the bottom layer. (Scale bar = 5 cm).
(DOCX)

S4 Fig Porous Teflon membrane modified with a layer of cured thiol-ene mixture. (A) During UV-exposure, regions on porous membrane that was protected by a plastic mask. These regions are indicated by the black arrow. When dry, the region appeared white and opaque. (B) Two chambers were wetted with DI water, as indicated by red arrows. Teflon membrane becomes transparent in visible light. (Scale bar = 5 mm).
(DOCX)

S5 Fig Burst pressure study for thiol-ene microchip. (a) Schematic view of the pressure system [48]. The thiol-ene microchip was clamped between the PC holders. The pressure sensor on the top of the PC holder will measure the pressure of the set-up. The syringes are compressed to provide the pressure into the microchip. (b) Microfluidic chip filled with red dye. The inlet and outlet ports for the bottom fluidic layer and outlet for the top layer were sealed with cured thiol-ene. The inlet port of the top fluidic layer is clamped between the mechanical device. (scale bar = 5mm).
(DOCX)

S6 Fig Phase contrast microscopic images of Caco-2 cells seeded in microchambers. (A) 2hrs after seeding before starting the continuous flow of DMEM across the cells; (B) 16hr after starting flow of DMEM across the cells. Images were taken at the same position of the same microchamber. (scale bar = 100μm).
(DOCX)

S7 Fig Phase contrast images of Caco-2 cells cultured in microchamber that was not functionalized with ECM. Images were taken at the same position of the microchamber. (A) Images of Caco-2 cells captured after 6hr of cell seeding; (B) Images of Caco-2 cells captured after 5 days of continuous perfusion. (Scale bar = 50μm).
(DOCX)

S8 Fig Overview of the entire microchamber of Caco-2 cells at day 8 of *in vitro* cell culture. Caco-2 cells showed very observable dark patches at regions close to the inlet of the microchamber (indicated by red arrows). Caco-2 cells displayed villous-like structures. (scale bar = 50 μm).
(DOCX)

S1 Table Tabulated data of the maximum pressure the different thiol-ene mixtures used for fabricating the microchips could withstand in different temperature conditions. All thiol-ene mixtures were prepared in stoichiometric ratios. Where 4T = tetra-thiol, 3T = tri-thiol and 3E = tri-allyl. (n = 6).
(DOCX)
