# Supplementary figures and images for: A multi-chamber microfluidic intestinal barrier model using Caco-2 cells for drug transport studies
Source: PLoS One. 2018 May 10;13(5):e0197101. doi: 10.1371/journal.pone.0197101 (PMC5944968; doi:10.1371/journal.pone.0197101)

**Supporting Information**

**
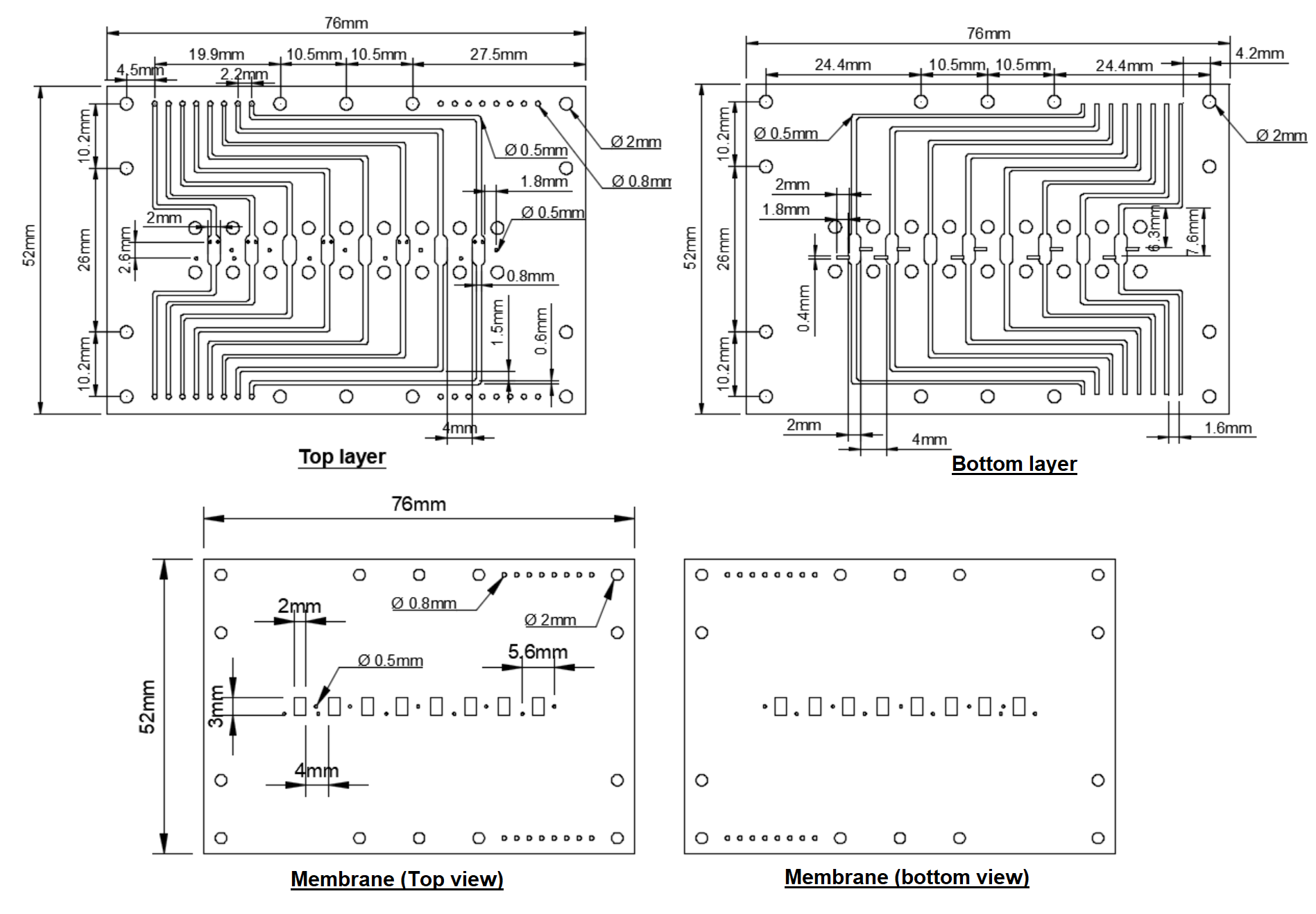
S1 Fig.** Cad drawings of the different layers in the thiol-ene microchip.

Supplement: S1 Fig — (DOCX) [file pone.0197101.s001.docx]
