# Supplementary material for: A multi-chamber microfluidic intestinal barrier model using Caco-2 cells for drug transport studies
Source: PLoS One. 2018 May 10;13(5):e0197101. doi: 10.1371/journal.pone.0197101 (PMC5944968; doi:10.1371/journal.pone.0197101)
Supplement: S2 Fig — (A) Schematic drawing of the microfluidic layers stacked together. (B) Enlarged view of the microchamber with the electrode ports. (DOCX) [file pone.0197101.s002.docx]

**Supporting Information**

(A)


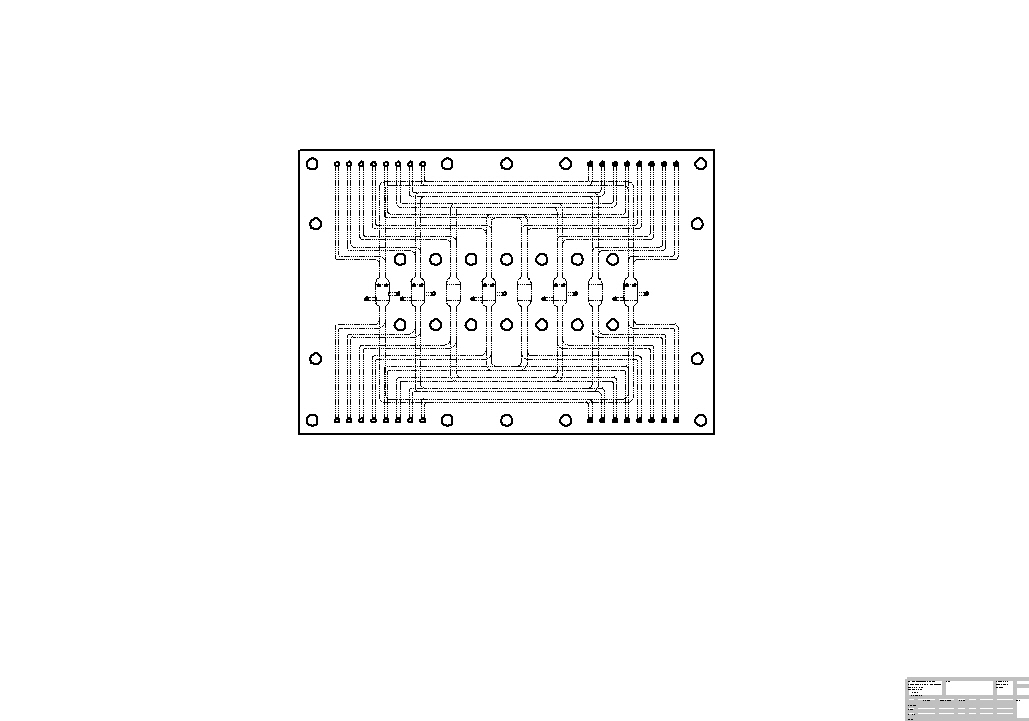


Top electrode

Bottom electrode

(B)

**S2 Fig.** Schematic overview of thiol-ene microchip with electrode ports and alignment markers. (A) Schematic drawing of the microfluidic layers stacked together. (B) Enlarged view of the microchamber with the electrode ports
