# Supplementary material for: A multi-chamber microfluidic intestinal barrier model using Caco-2 cells for drug transport studies
Source: PLoS One. 2018 May 10;13(5):e0197101. doi: 10.1371/journal.pone.0197101 (PMC5944968; doi:10.1371/journal.pone.0197101)
Supplement: S3 Fig — Blue arrow indicating the micropump perfusing through the top layer. Red arrow indicating the peristaltic micropump perfusing through the bottom layer. (Scale bar = 5 cm). (DOCX) [file pone.0197101.s003.docx]

**Supporting Information**


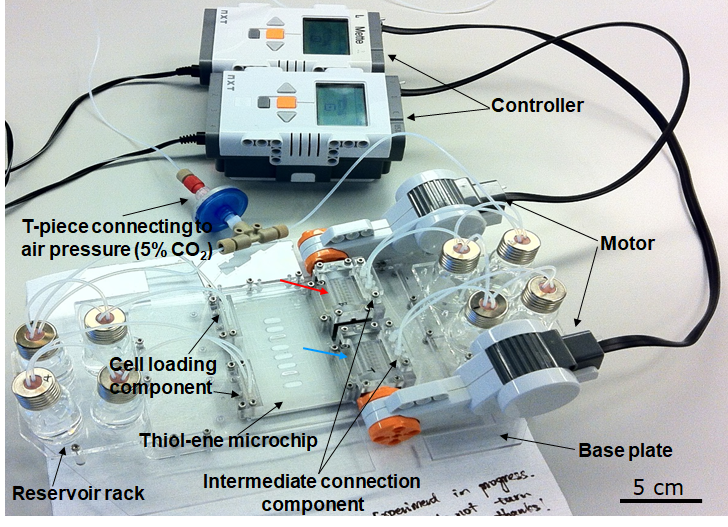


**S3 Fig**. Assembled thiol-ene microfluidicchip with the cell culture platform. Blue arrow indicating the micropump perfusing through the top layer. Red arrow indicating the peristaltic micropump perfusing through the bottom layer. (Scale bar = 5 cm)
