# Supplementary material for: A multi-chamber microfluidic intestinal barrier model using Caco-2 cells for drug transport studies
Source: PLoS One. 2018 May 10;13(5):e0197101. doi: 10.1371/journal.pone.0197101 (PMC5944968; doi:10.1371/journal.pone.0197101)
Supplement: S4 Fig — (A) During UV-exposure, regions on porous membrane that was protected by a plastic mask. These regions are indicated by the black arrow. When dry, the region appeared white and opaque. (B) Two chambers were wetted with DI water, as indicated by red arrows. Teflon membrane becomes transparent in visible light. (Scale bar = 5 mm). (DOCX) [file pone.0197101.s004.docx]

**Supporting Information**

(A)

(B)


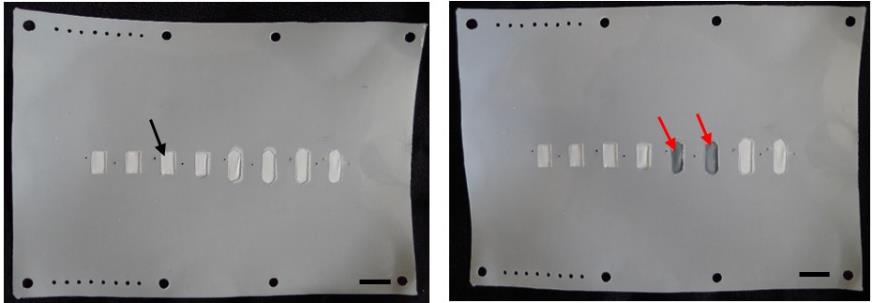


**S4 Fig.** Porous Teflon membrane modified with a layer of cured thiol-ene mixture. Black arrow indicating the region on porous membrane that was protected by a plastic mask during UV-exposure. When dry, the region appeared white and opaque. (b) Two chambers were wetted with DI water, as indicated by red arrows. Teflon membrane becomes transparent in visible light. (Scale bar = 5 mm).
