# Supplementary material for: A multi-chamber microfluidic intestinal barrier model using Caco-2 cells for drug transport studies
Source: PLoS One. 2018 May 10;13(5):e0197101. doi: 10.1371/journal.pone.0197101 (PMC5944968; doi:10.1371/journal.pone.0197101)
Supplement: S5 Fig — (a) Schematic view of the pressure system [48]. The thiol-ene microchip was clamped between the PC holders. The pressure sensor on the top of the PC holder will measure the pressure of the set-up. The syringes are compressed to provide the pressure into the microchip. (b) Microfluidic chip filled with red dye. The inlet and outlet ports for the bottom fluidic layer and outlet for the top layer were sealed with cured thiol-ene. The inlet port of the top fluidic layer is clamped between the mechanical device. (scale bar = 5mm). (DOCX) [file pone.0197101.s005.docx]

**Supporting Information**


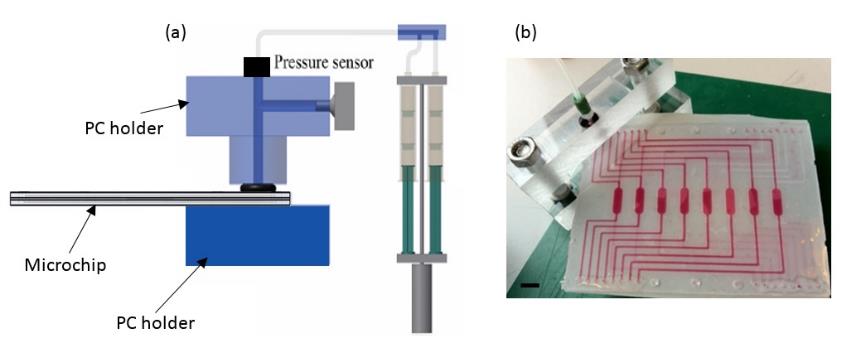


(A)

(B)

**S5 Fig.** Burst pressure study for thiol-ene microchip. (a) Schematic view of the pressure system ^1^. The thiol-ene microchip was clamped between the PC holders. The pressure sensor on the top of the PC holder will measure the pressure of the set-up. The syringes are compressed to provide the pressure into the microchip. (b) Microfluidic chip filled with red dye. The inlet and outlet ports for the bottom fluidic layer and outlet for the top layer were sealed with cured thiol-ene. The inlet port of the top fluidic layer is clamped between the mechanical device. (scale bar = 5mm)

**References**

1. Sikanen, T. M. *et al.* Fabrication and bonding of thiol-ene-based microfluidic devices. *J. Micromechanics Microengineering* **23,** 037002 (2013).
