# Supplementary material for: A multi-chamber microfluidic intestinal barrier model using Caco-2 cells for drug transport studies
Source: PLoS One. 2018 May 10;13(5):e0197101. doi: 10.1371/journal.pone.0197101 (PMC5944968; doi:10.1371/journal.pone.0197101)
Supplement: S6 Fig — (A) 2hrs after seeding before starting the continuous flow of DMEM across the cells; (B) 16hr after starting flow of DMEM across the cells. Images were taken at the same position of the same microchamber. (scale bar = 100μm). (DOCX) [file pone.0197101.s006.docx]

**Supporting Information**


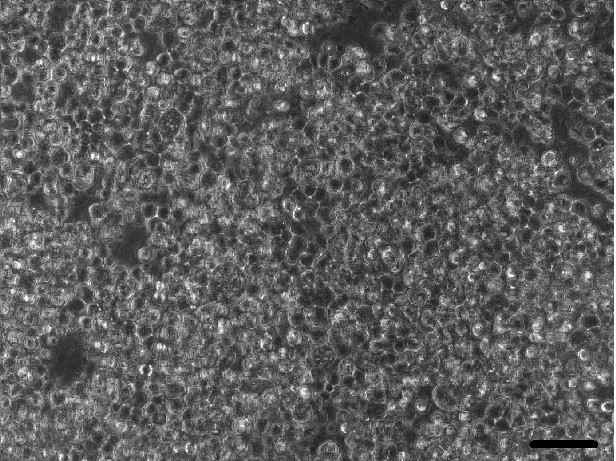

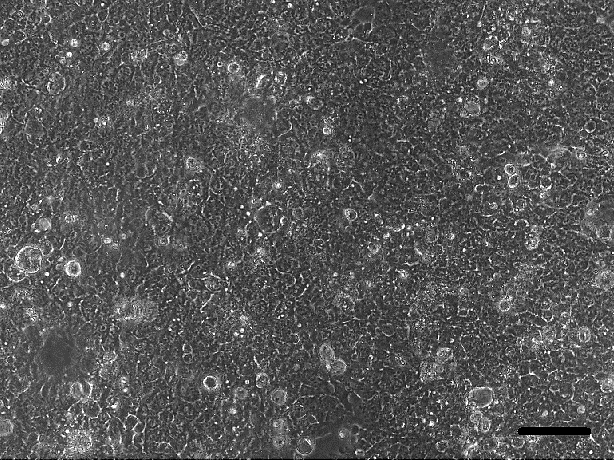


**(A)**

**(B)**

**S6 Fig.** Phase contrast microscopic images of Caco-2 cells seeded in microchambers (A) 2hrs after seeding before starting the continuous flow of DMEM across the cells; (B) 16hr after starting flow of DMEM across the cells. Images were taken at the same position of the same microchamber. (scale bar = 100µm)
