# Supplementary material for: A multi-chamber microfluidic intestinal barrier model using Caco-2 cells for drug transport studies
Source: PLoS One. 2018 May 10;13(5):e0197101. doi: 10.1371/journal.pone.0197101 (PMC5944968; doi:10.1371/journal.pone.0197101)
Supplement: S7 Fig — Images were taken at the same position of the microchamber. (A) Images of Caco-2 cells captured after 6hr of cell seeding; (B) Images of Caco-2 cells captured after 5 days of continuous perfusion. (Scale bar = 50μm). (DOCX) [file pone.0197101.s007.docx]

**Supporting Information**


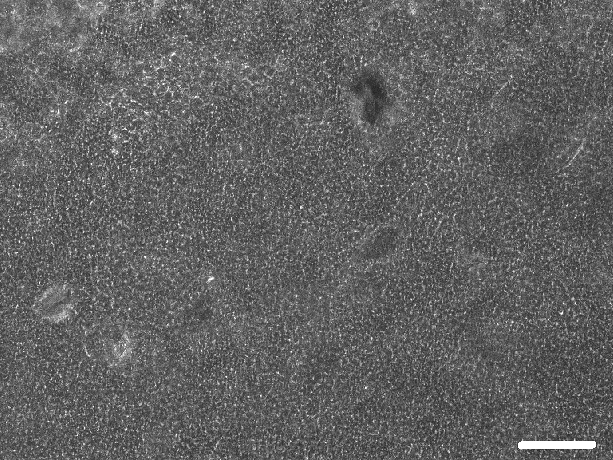

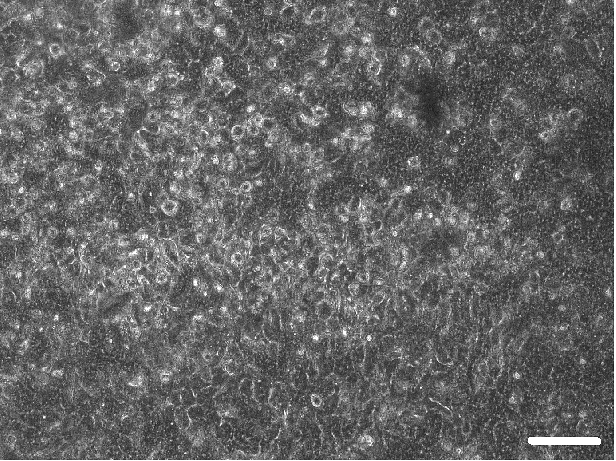


**(A)**

**(B)**

**Fig S7**. Phase contrast images of Caco-2 cells cultured in microchamber that was not functionalized with ECM. Images were taken at the same position of the microchamber. (A) Images of Caco-2 cells captured after 6hr of cell seeding; (B) Images of Caco-2 cells captured after 5 days of continuous perfusion. (Scale bar = 50µm)
