# Supplementary material for: A multi-chamber microfluidic intestinal barrier model using Caco-2 cells for drug transport studies
Source: PLoS One. 2018 May 10;13(5):e0197101. doi: 10.1371/journal.pone.0197101 (PMC5944968; doi:10.1371/journal.pone.0197101)
Supplement: S8 Fig — Caco-2 cells showed very observable dark patches at regions close to the inlet of the microchamber (indicated by red arrows). Caco-2 cells displayed villous-like structures. (scale bar = 50 μm). (DOCX) [file pone.0197101.s008.docx]

**Supporting Information**


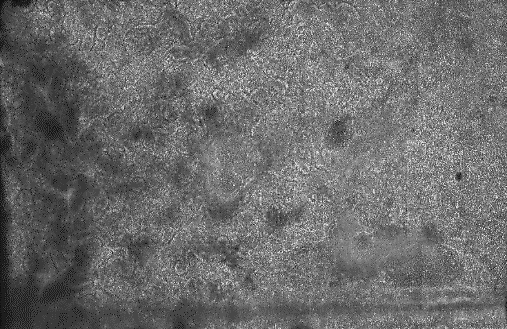


**S8 Fig.** Overview of the entire microchamber of Caco-2 cells at day 8 of *in vitro* cell culture. Caco-2 cells showed very observable dark patches at regions close to the inlet of the microchamber (indicated by red arrows). Caco-2 cells displayed villous-like structures. (scale bar = 50 µm)
