# Supplementary material for: A multi-chamber microfluidic intestinal barrier model using Caco-2 cells for drug transport studies
Source: PLoS One. 2018 May 10;13(5):e0197101. doi: 10.1371/journal.pone.0197101 (PMC5944968; doi:10.1371/journal.pone.0197101)
Supplement: S1 Table — All thiol-ene mixtures were prepared in stoichiometric ratios. Where 4T = tetra-thiol, 3T = tri-thiol and 3E = tri-allyl. (n = 6). (DOCX) [file pone.0197101.s009.docx]

| **Sample** | **Maximum pressure (Bars)** | **Temperature** |
| --- | --- | --- |
| 3T3E + 3T3E | 2.0 ± 0.66 | 25 °C |
| 4T3E + 4T3E | Layers could not be bonded |  |
| 4T3E + 3T3E | > 6.0 |  |
| 3T3E + 3T3E | < 0.3 | 37 °C |
| 4T3E + 4T3E | Layers could not be bonded |  |
| 4T3E + 3T3E | > 6.0 |  |

**S1 Table.** Tabulated data of the maximum pressure the different thiol-ene mixtures used for fabricating the microchips could withstand in different temperature conditions. All thiol-ene mixtures were prepared in stoichiometric ratios. Where 4T = tetra-thiol, 3T = tri-thiol and 3E = tri-allyl. (n = 6)
